# Structural and functional mapping of ion access pathways in the human K^+^-dependent Na^+^/Ca^2+^ exchanger NCKX2 using cysteine scanning mutagenesis, thiol-modifying reagents, and homology modelling

**DOI:** 10.1080/19336950.2025.2513268

**Published:** 2025-06-09

**Authors:** Robert T. Szerencsei, Shitian Cai, Hristina R. Zhekova, Ali H. Jalloul, D. Peter Tieleman, Paul P.M. Schnetkamp

**Affiliations:** a Department of Physiology & Pharmacology, Hotchkiss Brain Institute, Cumming School of Medicine, University of Calgary, Calgary, Canada; b Centre for Molecular Simulation, Department of Biological Sciences, University of Calgary, Calgary, Canada

**Keywords:** NCKX2, K^+^-dependent Na^+^/Ca^2+^ exchangers, homology modeling, molecular dynamics, ion access pathways

## Abstract

K^+^-dependent Na^+^/Ca^2+^ exchanger proteins (NCKX) are members of the CaCA superfamily with critical roles in vision, skin pigmentation, enamel formation, and neuronal functions. Despite their importance, the structural pathways governing cation transport remain unclear. To address this, we conducted a systematic study using cysteine scanning mutagenesis of human NCKX2 combined with the thiol-modifying reagents MTSET and MTSEA to probe the accessibility and functional significance of specific residues. We used homology models of outward-facing and inward-facing NCKX2 states and molecular dynamics (MD) simulations to compare and investigate residue accessibility in human NCKX2 based on the published structures of the archaeal NCK_Mj Na^+^/Ca^2+^ exchanger and the human NCX1 Na^+^/Ca^2+^ exchanger. Mutant NCKX2 proteins expressed in HEK293 cells revealed diverse effects of MTSET and MTSEA on Ca^2+^ transport. Of the 146 cysteine substitutions analyzed, 35 exhibited significant changes in Ca^2+^ transport activity upon treatment with MTSET, with 16 showing near-complete inhibition and six demonstrating increased activity. Residues within the cation binding sites and extracellular access channels were sensitive to modification, consistent with their critical role in ion transport, whereas intracellular residues showed minimal accessibility to MTSET but were inhibited by membrane-permeable MTSEA. Water accessibility maps from MD simulations corroborated these findings, providing a high-resolution view of water-accessible pathways. This study provides a comprehensive structural and functional map of NCKX2 ion access pathways, offering insights into the molecular basis of ion selectivity and transport. These findings highlight the key residues critical for cation binding and transport, advancing our understanding of the structural dynamics of NCKX2.

## Introduction

K^+^-dependent Na^+^/Ca^2+^ exchanger proteins (NCKX) belong to the CaCA super gene family of calcium/cation exchangers and are widely expressed in all animals [1–3]. The human *SLC24A* gene family consists of five different genes encoding NCKX1–5 proteins, which are important for a wide range of biological processes, including, but not limited to, the function of rod and cone photoreceptors in vision, human skin pigmentation, and enamel formation. Genetically modified mouse models have also demonstrated important roles in olfaction, melacortin-4-receptor mediated satiety and motor learning and memory (reviewed in [3–5]). K^+^-dependent Na^+^/Ca^2+^ exchanger proteins are bidirectional and obligatory coupled transporters operating in alternate access mode with a transport stoichiometry of four Na^+^ ions against one Ca^2+^ plus one K^+^ (reviewed in [6]). Within the CaCA gene family, NCKX proteins are closely related to K^+^-independent Na^+^/Ca^2+^ exchangers (NCX). The *SLC8A* gene family encodes human NCX1–3 proteins, which are widely expressed in all animals. Many prokaryotic and archaeal NCX-related sequences have been identified as well [1]. Several crystal structures have been obtained for the archaeal K^+^-independent Na^+^/Ca^2+^ exchanger found in *Methanococcus jannaschii*, NCX_Mj, and four distinct cation binding sites have been identified in the NCX_Mj structure [7,8]. The topology of the NCX_Mj protein observed in the crystal structure contains 10 transmembrane helices (TMH) divided into two sets of five TMH, separated by a short cytosolic loop. This topology has also been experimentally observed for human NCKX2 [9,10] and dog NCX1 [10,11]. Most recently, an identical topology was observed in the cryo-EM structures of the human cardiac NCX1 Na^+^/Ca^2+^ exchanger [12]. Although the topological arrangement of the 10 TMH is shared by NCX1 (containing 971 residues) and NCKX2 (containing 662 residues), sequence similarity is restricted to parts of TMH2–3 and TMH7–8 (~60 residues), which form the so-called α1 and α2 repeats and contain the 12 residues that make up the cation binding sites in the NCX_Mj and NCKX2 exchangers [5,8,13]. Previously, we used both alanine and cysteine scanning mutagenesis to generate a series of 193 residue substitutions that formed the core of the two alpha repeats [14,15]. In a series of previous studies, we used these mutant NCKX2 proteins expressed in HEK293 cells to identify residues that affect cation binding/transport. Substitution of these residues resulted in large changes in the K_m_ values for the substrate cations Na^+^ [15], K^+^ [16] and Ca^2+^ [17], respectively. A surprisingly large number of residue substitutions resulted in significant changes in the respective K_m_ values for all three cations involved, leading to both increases and decreases in K_m_ values. This was also the case for 11 of the 12 residues [16] that contributed to the four cation-binding sites identified in NCX_Mj [8] and in our NCKX2 homology model based on the NCX_Mj crystal structure [13]. Here, we carried out a systematic study of the accessibility of substituted cysteine residues in human mutant NCKX2 proteins to the hydrophilic thiol reagents MTSET (2-(trimethylammonium)ethyl methanethiosulfonate) and MTSEA (2-Aminoethyl methanethiosulfonate) as measured by the effect on NCKX2-mediated Ca^2+^ transport after transient transfection of the mutant proteins in HEK293 cells. The objective was to map out residues that line the access channels in the outward facing (OF) open and inward facing (IF) open states that allow substrate cations to permeate to and from the cation binding sites located well within the cell surface membrane in which NCKX2 is embedded. Molecular dynamics simulations using NCKX2 homology models that present the OF and IF open conformations of NCKX2 based on previously published structures of apo-NCX_Mj [7] and activated NCX1 [12], respectively, demonstrated good agreement with MTSET and MTSEA measurements and provided a three-dimensional reconstruction of the ion access pathways in NCKX2.

## Materials and methods

### Cell culture, transfection protocols, Ca^2+^ transport assay

Single residue cysteine substitutions were prepared in the short splice variant of human NCKX2 cDNA (AAF25811) with the c-myc tag (EQKLISEEDL) inserted between His83 and Gln84, as described previously [14,18]. The standard methodologies of subculturing HEK293 cells, transfection with WT and mutant NCKX2 subcloned in pcDNA3.1 (Invitrogen), were as described previously [19]. Measurement of NCKX2-mediated Ca^2+^ transport using the fluorescent Ca^2+^- indicating dyes Fluo4 and Fluo4FF was carried out in Na^+^-loaded HEK293 cells transfected with WT and mutant NCKX2 cDNAs, as described previously [20].

### MTSET or MTSEA pretreatment and effect on NCKX2-mediated transport

Reverse K^+^-dependent Na^+^/Ca^2+^ exchange transport was measured in Na^+^-loaded HEK293 cells suspended in a medium containing 150 mM LiCl, 0.1 mM EDTA, and 20 mM HEPES (adjusted to pH 7.4, with arginine). Measurements were carried out in a cuvette containing 2 mL of the above medium after the addition of 2 µM carbonyl cyanide p-trifluoromethoxyphenylhydrazone (FCCP) and 250 µM sulfinpyrazone under constant stirring in an SLM Series 2 Luminescence Spectrometer (SLM Instruments, Urbana, IL). MTSET ([2-(trimethylammonium) ethyl] methanethiosulfonate bromide) and MTSEA (2-Aminoethyl methanethiosulfonate) were added from a freshly prepared 100 mM stock solution to a final concentration of 2 mM. After 60 s, CaCl_2_ was added to a final concentration of 350 µM, followed by 10 s of adding a final concentration of 50 mM KCl to initiate NCKX2-mediated Ca^2+^ influx. After an additional 90 s, 0.1% saponin and 7.5 mM CaCl_2_ were added to permeabilize cells and to determine maximal Fluo4 or Fluo4FF fluorescence. Fluorescence was continuously recorded (excitation, 480 nm; bandwidth, 8 nm; emission, 530 nm; bandwidth, 8 nm) integrated over 200-ms time bins, and statistical analysis was carried out as described previously [17].

### Homology modelling of NCKX2

Two homology models of NCKX2, in the outward-facing open (OF NCKX2) and inward-facing open (IF NCKX2) states, were generated using the outward-facing apo-NCKX_Mj (PDB ID: 5HXH) and inward-facing Ca^2+^-bound activated NCX1 (PDB ID: 8SGT), respectively, as templates. Sequence alignment and protein modeling were performed using Modeller [21]. Because of the low sequence identity and for better reproduction of the transmembrane helices of the templates, the following constraints were imposed on OF NCKX2 during protein threading: alpha helical constraints between residues I135-K167, S171-A175, P493-T527, E533-L537, V598-I607, and F641-V648 and a 7 Å Cα-Cα distance between residues S205 and E651. Since the presence of a cysteine bond between C605 and C657 has been suggested previously [22], we introduced a Cys–Cys bond at these residues in our NCKX2 OF model. For IF NCKX2, the constraints were as follows: α-helical constraints between residues A134-G169, D173-F197, V203-R228, R240-W263, T271-R491, F499-T527, S531-R557, G561-F593, G602-W626, N629-R653, and a Cys–Cys bond between C605 and C657. Residues 291 to 485, which form a large intracellular regulatory domain in the NCKX2 protein [13] were removed from the models because of the absence of a template for their modeling. A thousand models were generated for each state with Modeller 10.5, an automodel function with the listed constraints. The DOPE [23] and GA341 [24] scores of the produced models were reviewed and 10–15 structures with the lowest DOPE and highest GA341 were selected for further evaluation by direct comparison with the templates and cross-reference of the position of known residues of importance for NCKX2 function. One final model was selected for each state for the Molecular Dynamics (MD) simulations.

### Molecular dynamics simulations of NCKX2

The homology models of OF NCKX2 and IF NCKX2 produced as described above were embedded in a lipid bilayer (with 150 molecules in the upper and lower leaflets) in a rectangular periodic box with dimensions of 107 × 107 × 101 Å, with 20 Å water layers on each side of the membrane and a 150 mm NaCl solution via CHARMM-GUI [25]. Since the MD simulations were geared at relaxation of the protein matrix in the presence of lipid, water, and ions and mapping of water permeation pathways through the protein center, a simplistic membrane made of POPC (an abundant lipid in the eukaryotic cells) was chosen for this study. A Cys–Cys bond patch was applied to C605-C657. Subsequently, the standard minimization and 6-step equilibration protocol of CHARMM-GUI with gradual release of positional restraints was performed, followed by 220 ns production runs with GROMACS 2022.6 [26], CHARMM36m force field (for proteins), CHARMM36 force field (for lipids, water, and ions), and the TIP3 water model [27–29]. The following settings were used during the production runs: 2 fs timestep, temperature of 310.15 K and pressure of 1 atm maintained with a Nose-Hoover thermostat [30] (t_t_ = 1.0) and a Parrinello-Rahman barostat [31] (semiisotropic coupling, t_p_ = 5.0), LINCS constraints for the bonds involving H-atoms [32], Particle Mesh Ewald algorithm for evaluation of the electrostatic interactions with r_coulomb_ 1,2 nm [33], and cutoff scheme for the VdW interactions with r_vdw_ 1.2 nm and r_vdw_ switch 1.0 nm). Water maps were generated from the MD trajectories using the VolMap tool of VMD 1.9.3 [34].

## Results and discussion

**Figure 1.**
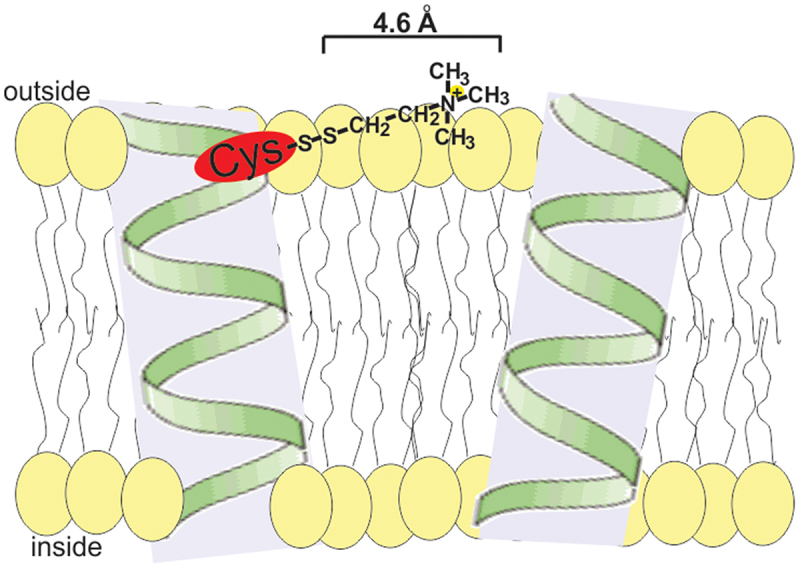
Cartoon representation of the effect of modifying a substituted cysteine residue by MTSET.

**Figure 2.**
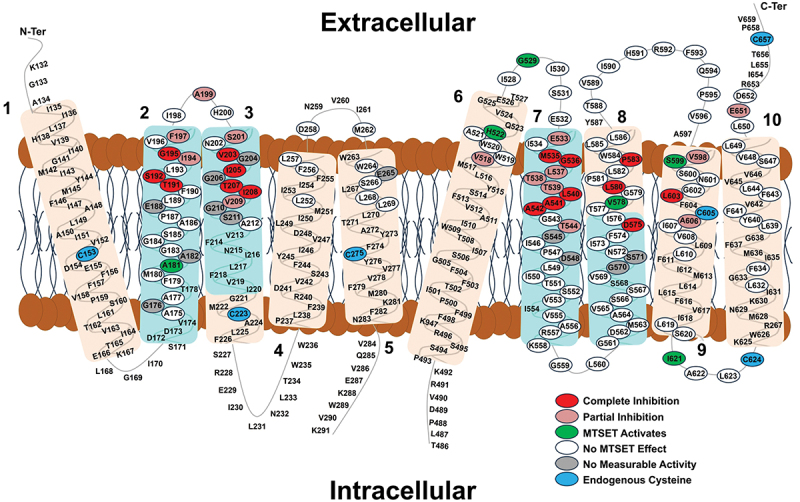
Schematic representation of the human NCKX2 topology highlighting the different residue groups that showed shifts in Ca^2+^ transport activity when treated with MTSET and compared with WT NCKX2.

The mature human WT NCKX2 protein (after cleavage of the signal peptide) contains seven endogenous cysteine residues, six of which are located within the 10 transmembrane helices (TMH) and short connecting loops, while the seventh is in the middle of a large hydrophilic loop located in the cytosol Figure 2. The principle of the accessibility assay is illustrated in Figure 1. An accessible cysteine residue in the NCKX2 protein can form a thiol bond with the hydrophilic thiol reagent MTSET and add a 4.6 Å positively charged spacer that could impede the passage of cations if located in the selective Ca^2+^, K^+^ and Na^+^ access channel leading to the cation binding sites found toward the center of the membrane. The positively charged MTSET could impede NCKX2-mediated transport via charge-specific repulsion due to its positive charge, via steric hindrance of the cation access to the cation coordinating residues deep into the protein interior or via structural modulation leading to entrapment of the transporter in a state that either boosts or impairs transport depending on the position of the substituted residue. As MTSET is membrane-impermeable, and our assay measures Ca^2+^ influx via reverse exchange, our MTSET results most likely only address the OF open conformation of the NCKX2 protein and the OF conformation of the ion transport pathway. Treatment of HEK293 cells expressing WT NCKX2 with 2 mm MTSET for periods up to 10 min had no effect on NCKX2-mediated Ca^2+^ influx via the so-called reverse K^+^-dependent Na^+^/Ca^2+^ exchange observed as an increase in Fluo4FF fluorescence. We used a cuvette-based assay to measure Ca^2+^ influx via reverse K^+^-dependent Na^+^/Ca^2+^ exchange in Na^+^-loaded HEK293 cells transiently transfected with WT or mutated NCKX2 cDNA and loaded with the fluorescent Ca^2+^-indicating dye Fluo4 or Fluo4FF [20]. The lack of an effect of MTSET on Ca^2+^ transport implies that either the endogenous cysteine residues were not accessible to the MTSET reagent or that modification by MTSET and introducing a positive charge on the substituted cysteine residue had no effect on K^+^-dependent Na^+^/Ca^2+^ exchange transport. Over the years, we have created a collection of mutant NCKX2 proteins representing 146 separate cysteine substitutions, and these mutant NCKX2 proteins displayed a wide range of transport activities relative to WT NCKX2. Therefore, we used two different Ca^2+^-indicating dyes with different affinities that allowed us to accommodate both WT NCKX2 and mutant NCKX2 proteins with transport activities as low as 2% of that of WT [15]. Fluo4 (K_d_ 0.35 µM) was used to measure mutants with poor activity, whereas Fluo4FF (K_d_ 9.7 µM) was used to measure mutants with activities closer to WT NCKX2.

### MTSET results in modification of reverse K^+^-dependent Na^+^/Ca^2+^ exchange transport in a subset of inserted cysteine substitution mutant NCKX2 proteins

We expressed each of the above 146 single residue substitution mutant NCKX2 proteins separately in HEK293 cells and examined the effect of MTSET on Ca^2+^ influx via reverse K^+^-dependent Na^+^/Ca^2+^ exchange, again observed as an increase in Fluo4FF or Fluo4 (in case of mutant NCKX2 proteins with reduced transport activity) fluorescence. Like WT NCKX2, for 98 of the cysteine substitutions, the application of MTSET caused no significant change in the rate of Ca^2+^ influx (Figure 2, white circles). In the case of 12 cysteine substitutions, the mutant NCKX2 proteins showed no measurable Ca^2+^ transport activity (<1% of WT NCKX2) (Figure 2, gray circles). Six of these residue substitutions (G184C, E188C, S211C, S545C, D548C, and S571C) feature residues that belong to the four cation binding sites where sensitivity to mutagenesis is expected. These residues are conserved in human NCKX1–4. The other six residue substitutions that yielded nonfunctional NCKX2 proteins (G176C, A182C, G204C, G206C, G210C, and E265C) were conserved in human NCKX1–4 as well, again highlighting the functional importance of these residues. Previously, we have shown that lack of transport activity of these 12 residue substitutions was not caused by lack of protein expression of lack of trafficking to the plasm membrane [14].

Highlighted in *white* are cysteine substitutions of residues that show no significant MTSET effect on Ca^2+^ transport activity. Highlighted in *red* are cysteine substitutions of residues that resulted in greater than 75% inhibition of Ca^2+^ transport activity when treated with MTSET. Highlighted in *pink* are cysteine substitutions of residues that resulted in 25–75% inhibition of Ca^2+^ transport activity when treated with MTSET. Highlighted in *green* are cysteine substitutions of residues that result in increased Ca^2+^ transport activity when treated with MTSET. Highlighted in *grey* are cysteine substitutions of residues that resulted in total loss of Ca^2+^ transport activity (<1% of WT NCKX2). The endogenous cysteine residues are highlighted in *blue*.

**Figure 3.**
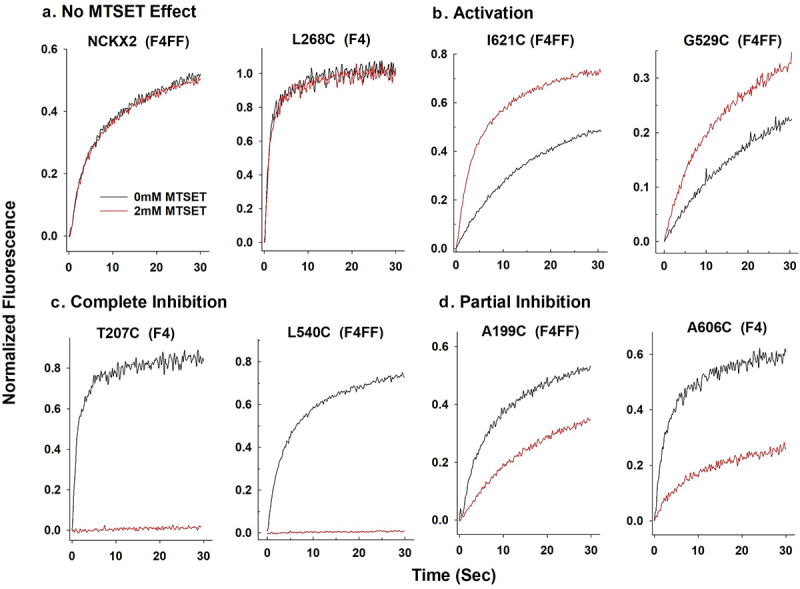
Examples of the different effects of MTSET on Ca^2+^ transport activity of mutant human NCKX2 proteins carrying single residue cysteine substitutions and expressed in HEK293 cells.

**Figure 4.**
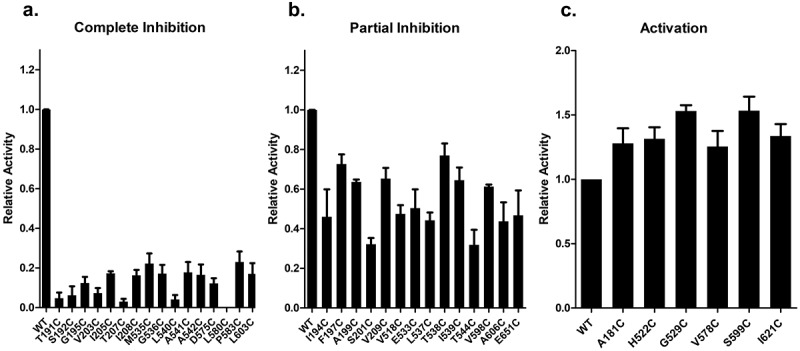
Bar graph representing the effect of MTSET on the Ca^2+^ transport activity of the different NCKX2 single residue cysteine substitutions of the WT-NCKX2 clone.

For 35 of the cysteine substitutions the mutant NCKX2 proteins were affected in a statistically significant manner and examples of four different activity profiles are illustrated in Figure 3 (please note the two different Ca^2+^-indicating dyes used to accommodate both WT-like mutants and mutants with strongly reduced Ca^2+^ transport activities). Figure 4 presents bar diagrams for all mutant NCKX2 proteins, where the reaction with MTSET produced statistically significant changes in the Ca^2+^ transport activity. Near-complete (>75%) inhibition was observed for 16 mutants (Figure 4(a), red circles in Figure 2), and 14 of these 16 mutated residues were conserved in human NCKX1–4. Partial inhibition (between 30% and 75%) of Ca^2+^ influx was observed in 14 mutated residues (Figure 4(b), pink circles in Figure 2), and seven of these 14 residues were conserved in NCKX1–4. For six of the cysteine substitutions, the mutant NCKX2 proteins showed an increase in the rate of Ca^2+^ influx (Figure 4(c), green circles in Figure 2), and three of the six were conserved in human NCKX1–4.

Traces showing changes in intracellular Ca^2+^ concentration in HEK293 cells detected by either Fluo4 or Fluo4FF as indicated. HEK293 cells expressing WT-NCKX2 or mutant NCKX2 proteins carrying a single residue substitution to cysteine were loaded with either Fluo4-AM or Fluo-4FF-AM. Next, transfected HEK293 cells were treated with 0 or 2 mM MTSET for 60 s before initiating Ca^2+^ influx via reverse exchange; Ca^2+^ influx was initiated by the addition of 350 µM CaCl_2_ and the addition of 50 mM KCl at time zero. *Panel A* represents the group of residue substitutions that showed no MTSET effect on Ca^2+^ transport activity (WT-NCKX2 and L268C). *Panel B* represents the group of residue substitutions that showed an increase in Ca^2+^ transport activity upon treatment with 2 mM MTSET (L621C and G529C). *Panel C* represents the group of residue substitutions that completely inhibited Ca^2+^ transport activity when treated with 2 mM MTSET (T207C and L540C). *Panel D* represents the group of residue substitutions that showed partial inhibition of Ca^2+^ transport activity upon treatment with 2 mM MTSET (A199C and A606C). Temperature: 25 ºC.

Panel A. represents “Group I:” 16 single residue cysteine substitutions which resulted in greater than 75% inhibition of transport activity compared with WT-NCKX2 exchanger. *Panel B* represents “Group II:” 15 single-residue cysteine substitutions with transport activity between 25% and 75% of the WT-NCKX2 exchanger. *Panel C* represents “Group III:” six single-residue cysteine substitutions, which resulted in an increased transport activity in comparison to the WT-NCKX2 exchanger. Experiments were replicated between three and ten times, and comparison against the WT-NCKX2 was carried out using a two-tailed *t*-test; values represent the means ± SEM.

### Location of the inserted cysteine residues within the NCKX2 topology and the effect of MTSET

Figure 2 illustrates the location of 146 residues investigated in our topological model of the OF NCKX2 protein [9,10]. Clearly, alpha repeat residues that are located near the extracellular space (i.e. the top halves of TMH2, TMH3, and TMH7 in Figure 2) represent two clusters of residues that are most strongly affected by MTSET, whereas the alpha repeat residues that are located near the intracellular space (i.e. the bottom halves of TMH2, TMH7, and TMH8 in Figure 2) are not affected by MTSET, as this hydrophilic and positively charged reagent is unlikely to have access to these substituted cysteine mutants, consistent with our topology model.

**Figure 5.**
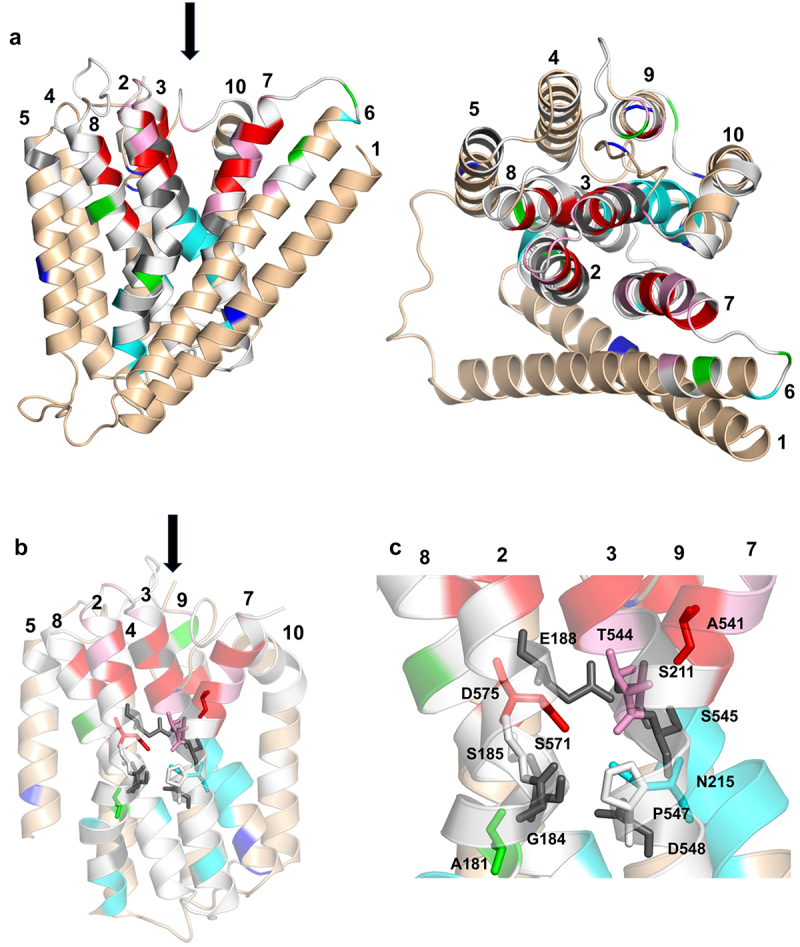
Homology model of OF human NCKX2 based on the X-ray crystal structure of the archaebacterial NCX_Mj protein, illustrating the results from the MTSET measurements.

Figure 5(a) illustrates our homology model of the OF conformation of the human NCKX2 protein in front and top views. The color coding of the helices follows the colors displayed in Figure 2. Figures 5(b,c) show the binding cavity of OF NCKX2 with the location of the 12 residues that form the cation-binding sites. The arrows indicate the location and direction of the ion-access pathway in the OF NCKX2. A more detailed breakdown of the MTSET results projected onto the OF NCKX2 structure is shown in Figure S1. A water map from the MD simulations of OF NCKX2, which indicates the depth of penetration of water (and, therefore, the MTSET probe) within the protein matrix, is shown in Figure S2 (left). Of the cation binding site residues that are in the water-accessible part of the access channel (i.e. closer to the extracellular surface, S185, E188, S211, T544, A541, S545, P547, and D575), three cysteine substitutions had no transport activity (E188C, S211, and S545C), A541C showed ~80% inhibition, T544C showed ~70% inhibition, and D575C showed ~90% inhibition. The S185C and P547C mutants were not affected by MTSET. Because the water map (Figure S2) shows access of water to the depths of S185C and P547C, the lack of effect is most likely because they face away from the ion access cavity and toward the interhelical space (Figure 5(c)).

Panel A. Front and top views of OF NCKX2. Panel B. Binding cavity of the OF NCKX2. Residues from the cation-binding site are shown as sticks. TMH 1 and 6 have been removed for clarity. Panel C. Close-up view of the binding site of OF NCKX2. The colors of the helices and residues are consistent with those shown in Figure 2. The position of the entry/exit of the ion-access pathway is indicated by an arrow.

The MTSET mapping results illustrated in Figure 2 are consistent with our model of NCKX2 topology; however, with two notable exceptions: the activation of transport activity by MTSET observed for the A181C and I621C (see Figures 2 and 3) mutant NCKX2 proteins. A181 is one of the twelve residues that form the four cation binding sites, and the residue is thought to be located at the bottom of the binding area and closest to the intracellular surface, while A541 is at the top of the four cation binding sites and closest to the extracellular surface. Thus, both may act as “gates” that open and close the ion transport pathway [13]. The A541C mutant, as expected in such a configuration, results in strong inhibition of Ca^2+^ transport by MTSET, while the A181C mutant is surprisingly activated by MTSET. In our topology model, I621C is located in TM9 near the cytosolic surface, and, as with the activation of the A181C mutant by MTSET, we do not have a ready explanation for its activation by MTSET. Possible scenarios that may explain the observed MTSET activation in these residues include altered inter-helical packing or interaction with the lipids at the protein – lipid interface in the vicinity of these residues or, possibly, allosteric effects of the MTSET probe bound in these positions, especially in the case of A181. The explanation of the mechanism of MTSET activation requires additional modeling and experimental work outside the scope of the current exploratory study.

### Probing intracellular accessibility of inserted cysteine residues in the IF state of NCKX2 with MTSEA

**Figure 6.**
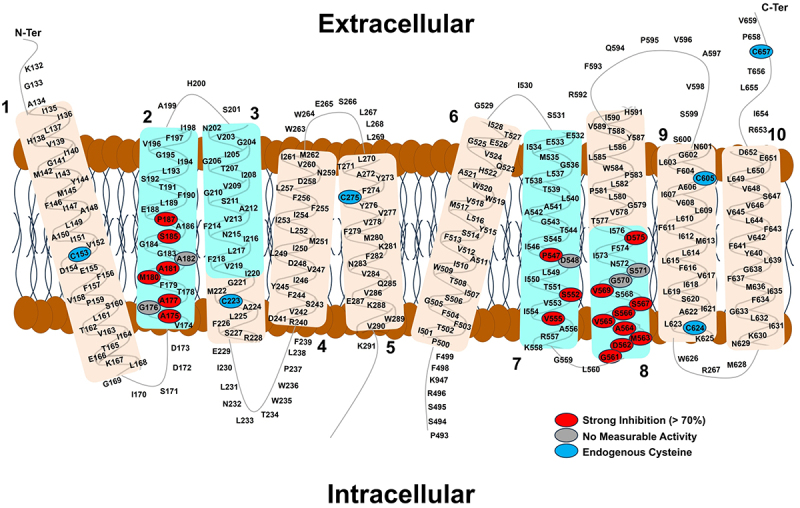
Schematic representation of the human NCKX2 topology highlighting the different residue groups that showed shifts in Ca^2+^ transport activity when treated with MTSEA and compared with WT NCKX2.

**Figure 7.**
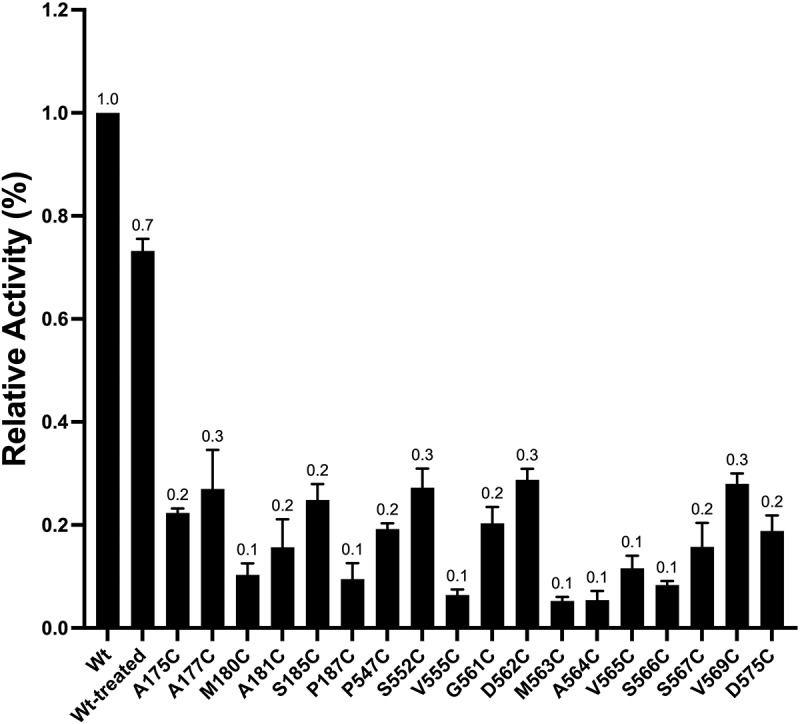
Bar graph representing the effect of MTSEA on the Ca^2+^ transport activity of the different single residue cysteine substitutions of the WT-NCKX2 clone. The bars represent the effect of MTSEA on transport activity (normalized to WT NCKX2 transport activity) of WT NCKX2 and 18 different cysteine substitution mutants. Experiments were replicated between 3 and 6 times, 11 times for the WT NCKX2. Comparison against the WT-NCKX2 activity was carried out using a two-tailed *t*-test; values represent the means ± SEM.

To address the accessibility of cation-binding residues that are thought to be located near the intracellular surface and at the bottom of the cation binding sites, we carried out a smaller survey using the membrane-permeant reagent MTSEA (2-Aminoethyl methanethiosulfonate) and its effect on the Ca^2+^ transport activity of WT and cysteine-substituted residues with a location in the bottom half of the alpha repeats (the bottom halves of TMH2, TMH7, and TMH8). Compared to MTSET, MTSEA lacks the three methyl groups on the nitrogen atom. Consequently, it is smaller and does not carry a fixed positive charge. Although predominantly protonated at physiological pH, the unprotonated and uncharged form can readily cross biological membranes and access residues from the intracellular space in addition to accessing residues from the extracellular space. The caveat is that MTSEA resulted on average in ~30% inhibition of Ca^2+^ transport in WT NCKX2; therefore, we limited the analysis to the 18 cysteine substitution mutants for which MTSEA caused a greater than 70% inhibition of Ca^2+^ transport. Figure 6 illustrates the location of these 18 cysteine substitution mutants and Figure 7 shows the bar diagram of the inhibition pattern for these 18 residues as well as that for WT NCKX2. The topology of the TMH differs between Figures 2 and 6 because of the different templates used for OF and IF NCKX2 (NCX_Mj and NCX1, respectively). However, the positions of the α-repeats and the residues from the cation-binding sites at the protein center were consistent in both models. Of the cysteine substitutions tested with MTSEA, the D575C mutant NCKX2 protein was the only one that was inhibited by both MTSEA and MTSET. Ca^2+^ transport of 6 cysteine substitutions in the bottom half of TMH2 (part of α-repeat 1) was strongly inhibited by MTSEA; all 6 residues are conserved between NCKX1–4. The Ca^2+^ transport of 12 additional cysteine substitutions in the bottom halves of TMH7 and TMH8 (part of α-repeat 2) was strongly inhibited by MTSEA; 10 of these 12 residues are conserved between NCKX1–4. Again, these results are consistent with the critical importance of NCKX2 α-repeats 1 and 2 in accommodating cation-binding residues as well as the access channel to the binding sites.

Residues that exhibited >70% inhibition of Ca^2+^ transport activity when treated with MTSEA are highlighted in *red*. Highlighted in *grey* are residues that exhibited no measurable transport activity of the mutant substituted cysteine NCKX2 protein. Endogenous cysteine residues are highlighted in *blue*.

**Figure 8.**
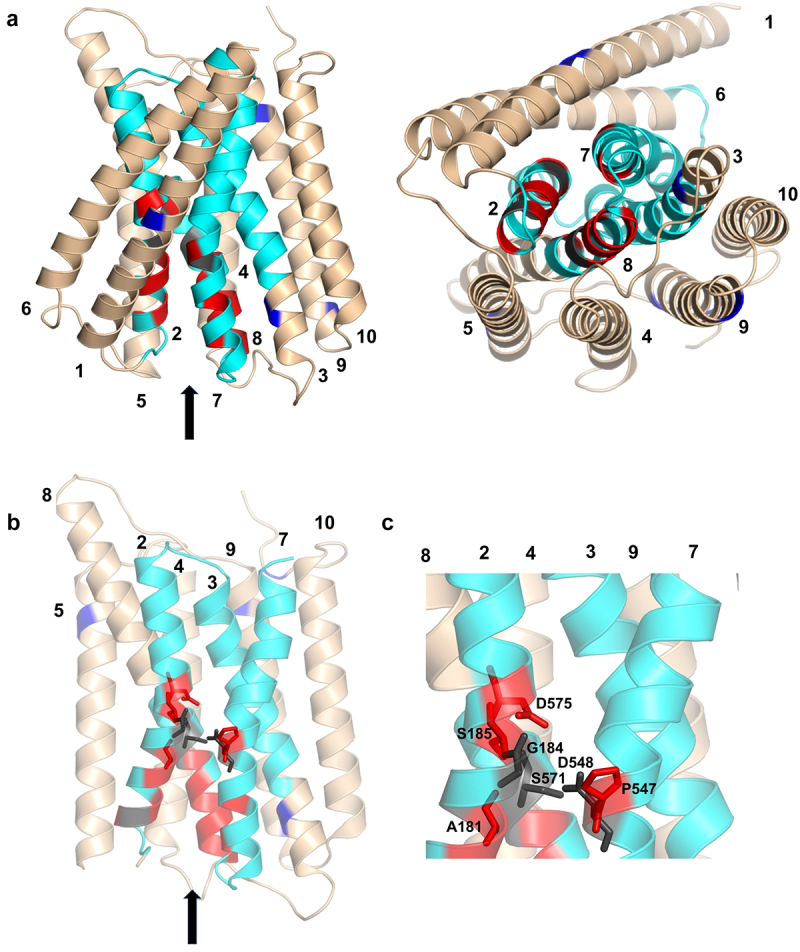
Homology model of IF human NCKX2 based on the cryoEM structure of the human Ca^2+^ bound activated NCX1 protein, illustrating the results from the MTSEA measurements.

Figure 8 displays the IF model of NCKX2 (side and bottom views), the binding cavity flanked by the α-repeats, and a close-up of the residues from the binding sites. The colors of the helices and residues correspond to those shown in Figure 6. A more detailed breakdown of the MTSEA results projected onto the IF NCKX2 structure is shown in Figure S3. A water map from MD simulations of the IF NCKX2 model is shown in Figure S2 (right) and indicates the access of water (and, therefore, MTSEA probes) to the entire binding site, as shown in Figure 8(c).

Panel A. Front and bottom views of IF NCKX2. Panel B. Binding cavity of IF NCKX2. Residues from the cation-binding site are shown as sticks. TMH 1 and 6 have been removed for clarity. Panel C. Close-up view of the IF NCKX2 binding site. The colors of the helices and residues are consistent with those shown in Figure 7. The position of the entry/exit of the ion-access pathway is indicated by an arrow.

**Figure 9.**
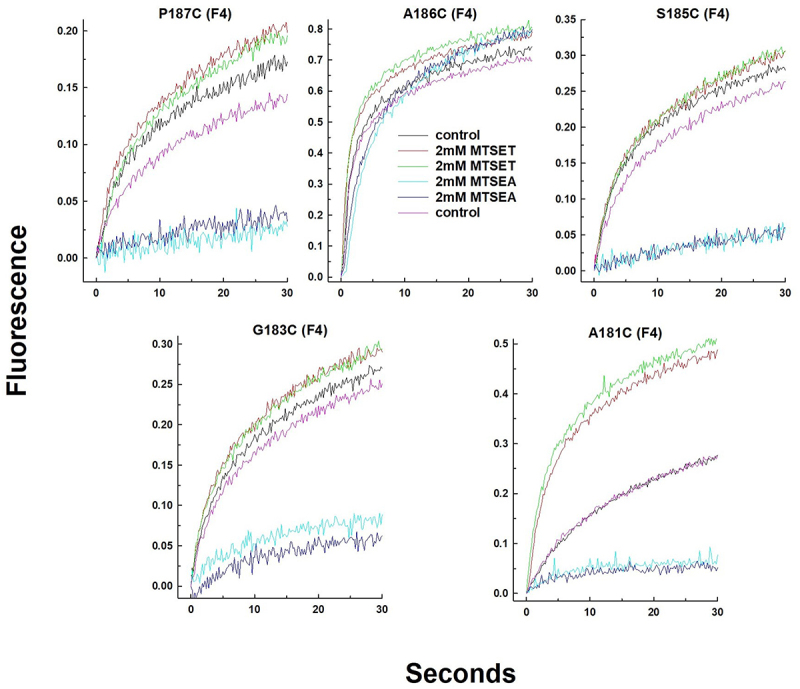
The effect of MTSET and MTSEA on the transport activity of the NCKX2 A181C substitution and cysteine substitutions of immediate neighboring residues.

Notably, the A181C mutant NCKX2 protein was inhibited by MTSEA (Figure 6) but activated by MTSET (Figure 2). We performed experiments in which we compared the effects of MTSEA and MTSET on the transport activity of the A181C NCKX2 mutant protein as well as NCKX2 mutant proteins, representing four additional residues of the α-1 repeat that are close to A181 and are likely located in the bottom half of TMH1. One such experiment is illustrated in Figure 9, which shows that MTSET had little effect on Ca^2+^ transport mediated by G183C, S185C, A186C, and P187C mutant NCKX2 proteins, unlike its activating effect on the A181C mutant. In contrast, MTSEA strongly inhibited the A181C, G183C, S185C, and P187C mutant NCXK2 proteins but had little effect on transport mediated by the A186C mutant NCKX2 protein. These results are consistent with the importance of these residues in transport mediated by the IF state of the NCKX2 protein, and with the water map of the IF state (Fig. S2). The strong inhibitory effect of MTSEA on the A181C mutant NCKX2 protein parallels the inhibitory effect of MTSET on the A541C mutant NCKX2 protein. Both residues are thought to act as the top and bottom gates of the transport pathway, respectively, and the reaction with the MTSEA and MTSET reagents is expected to impede cation transport.

Traces showing changes in intracellular Ca^2+^ concentration as measured by Fluo4 in HEK293 cells expressing mutant NCKX2 proteins carrying the indicated cysteine substitutions. The experimental protocol is described in Figure 3 legend. MTSET and MTSEA were added from a 100 mM stock solution to a final concentration of 2 mM prior to the initiation of Ca^2+^ influx at time zero, as described in the legend of Figure 3. Fluo4 was used as the Ca^2+^-indicating dye for all five mutants. Temperature: 25°C.

## Supplementary Material

Supplemental Material

## Data Availability

The data that support the findings of this study are available from the corresponding author, [PPMS], upon reasonable request.
