# Supplementary material for: Structural and functional mapping of ion access pathways in the human K+-dependent Na+/Ca2+ exchanger NCKX2 using cysteine scanning mutagenesis, thiol-modifying reagents, and homology modelling
Source: Channels (Austin). 2025 Jun 9;19(1):2513268. doi: 10.1080/19336950.2025.2513268 (PMC12150658; doi:10.1080/19336950.2025.2513268)
Supplement: Supplemental Material [file KCHL_A_2513268_SM9013.zip › Supplementary figures/Supplementary figure captions.docx]

**FigureS1. MTSET results projected on OF NCKX2**

Panel A. Residues where MTSET leads to complete inhibition (red sticks). Panel B. Residues where MTSET leads to partial inhibition (pink sticks). Panel C. Residues where MTSET leads to activation (green sticks). Panel D. Residues where there is no effect on transport by mutation to Cys and MTSET (white sticks). Panel E. Residues where mutation to Cys leads to an inactivated protein (gray sticks). Panel F. Endogenous Cys residues (blue sticks).

**Figure S2. Water maps (blue mesh) in the OF and IF NCKX2 obtained from MD simulations.**

The helices and residues are colored consistent with Figures 2 and 5 (OF NCKX2 map) and Figures 6 and 8 (IF NCKX2 map).

**FigureS3. MTSEA results projected on IF NCKX2**

Panel A. Residues where MTSEA leads to strong (> 50%) inhibition (red sticks). Pane B. Endogenous Cys residues (blue sticks). Panel C. Residues where mutation to Cys leads to an inactivated protein (gray sticks).
