# Supplementary figures and images for: Structural and functional mapping of ion access pathways in the human K+-dependent Na+/Ca2+ exchanger NCKX2 using cysteine scanning mutagenesis, thiol-modifying reagents, and homology modelling
Source: Channels (Austin). 2025 Jun 9;19(1):2513268. doi: 10.1080/19336950.2025.2513268 (PMC12150658; doi:10.1080/19336950.2025.2513268)

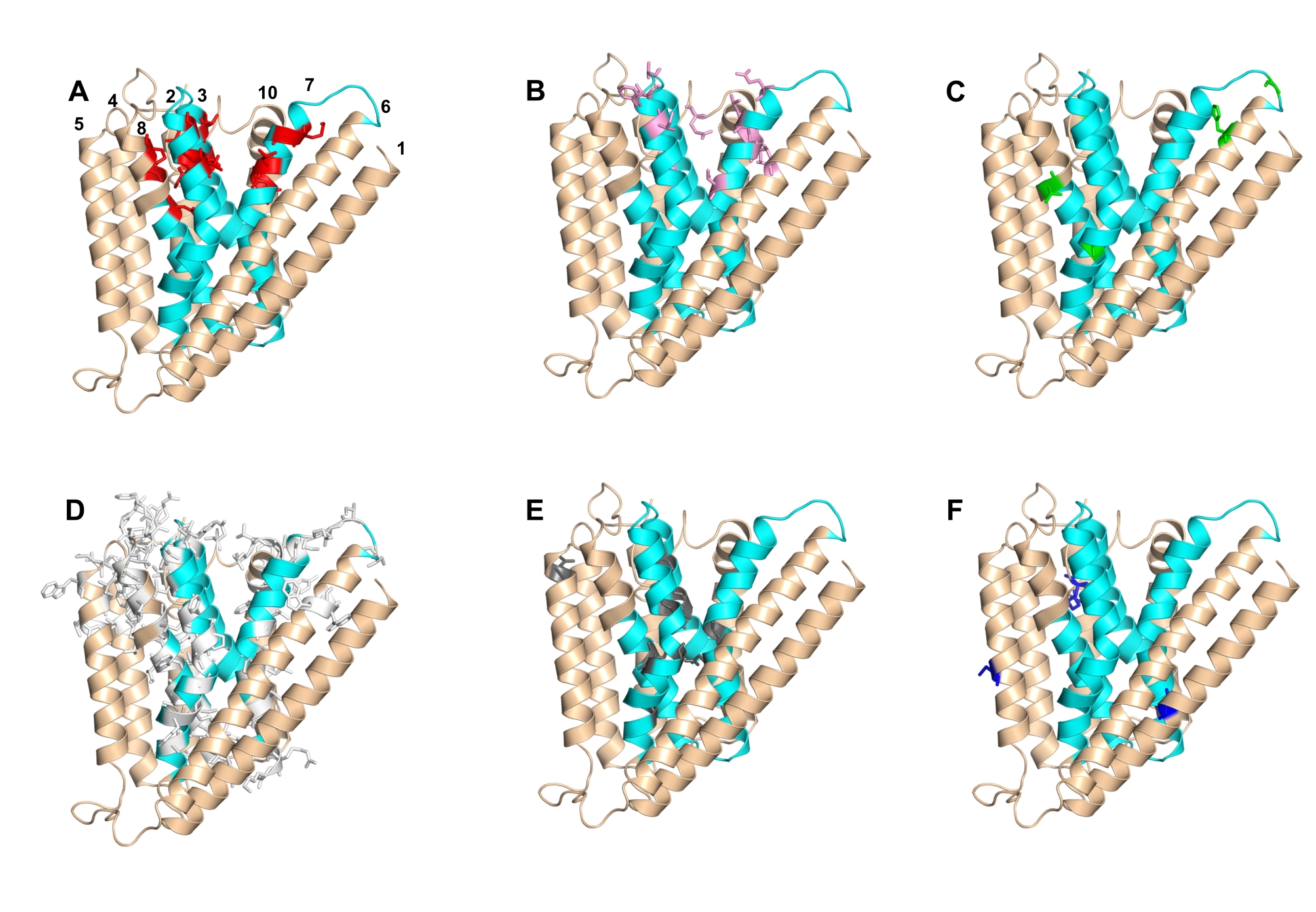

Supplement: Supplemental Material [file KCHL_A_2513268_SM9013.zip › Supplementary figures/Szerencsei et al Figure S1.jpg]

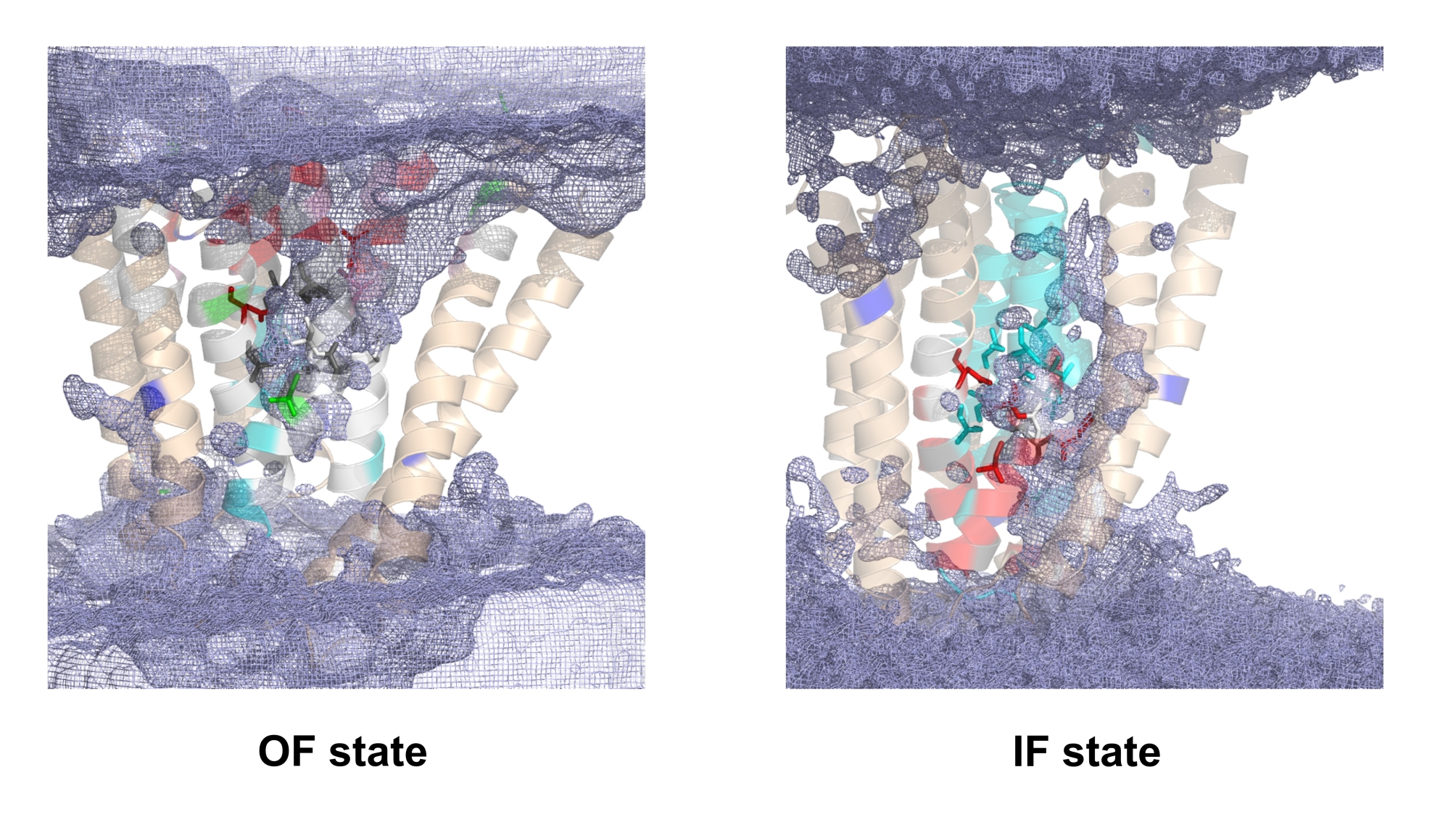

Supplement: Supplemental Material [file KCHL_A_2513268_SM9013.zip › Supplementary figures/Szerencsei et al Figure S2.jpg]

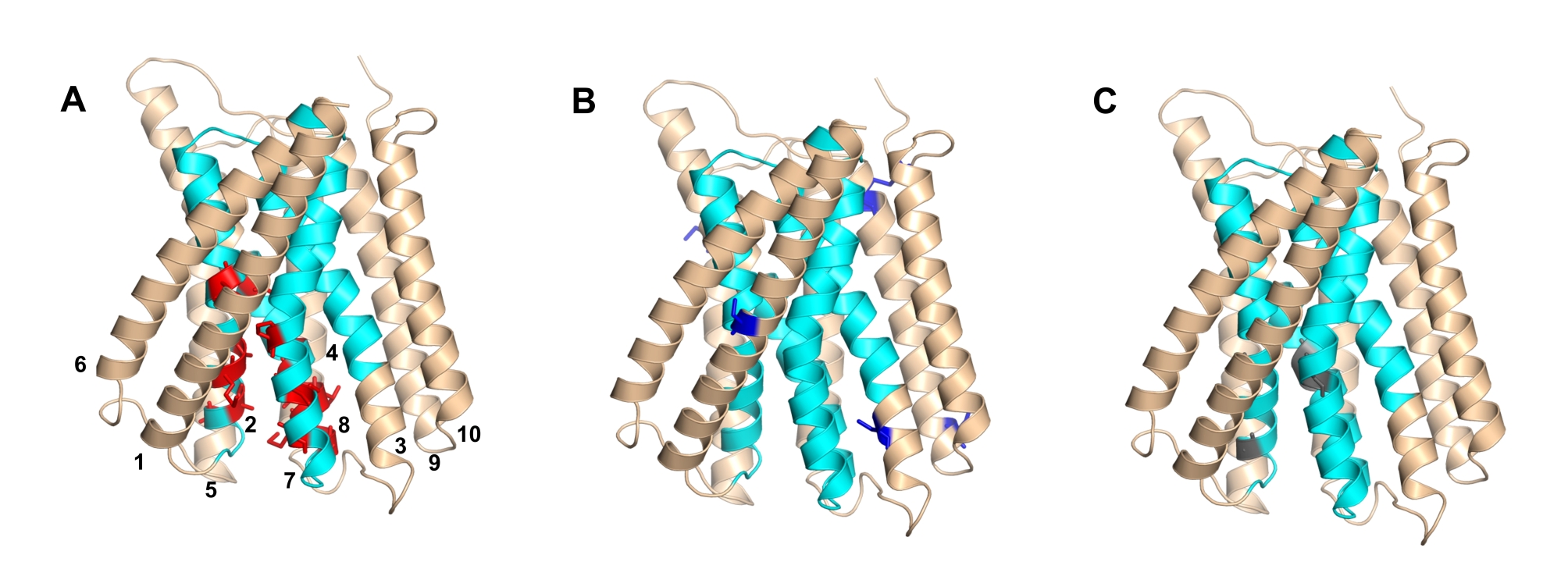

Supplement: Supplemental Material [file KCHL_A_2513268_SM9013.zip › Supplementary figures/Szerencsei et al Figure S3.jpg]
